# Optimized Synthesis of Biodegradable Elastomer PEGylated Poly(glycerol sebacate) and Their Biomedical Application

**DOI:** 10.3390/polym11060965

**Published:** 2019-06-03

**Authors:** Yanxiang Wang, Haiwa Wu, Zihao Wang, Jingjing Zhang, Jing Zhu, Yifan Ma, Zhaogang Yang, Yuan Yuan

**Affiliations:** 1 Engineering Research Center for Biomaterials of Ministry of Education, East China University of Science and Technology, Shanghai 200237, China; wangyanxiangwyz@163.com (Y.W.); zh931212@163.com (Z.W.); 2 Key Laboratory for Ultrafine Materials of Ministry of Education, East China University of Science and Technology, Shanghai 200237, China; 3 Department of Pathology, College of Medicine, The Ohio State University, Columbus, OH 43210, USA; wu.680@osu.edu; 4 Department of Chemical and Biomolecular Engineering, The Ohio State University, Columbus, OH 43210, USA; zhang.8211@osu.edu; 5 Department of Pharmaceutics, College of Pharmacy, The Ohio State University, Columbus, OH 43210, USA; syusejing@gmail.com; 6 Department of Radiation Oncology, The University of Texas Southwestern Medical Center, Dallas, TX 75390, USA

**Keywords:** poly(glycerol sebacate) (PGS), polyethylene glycol (PEG), biomedical application, biodegradable elastomer

## Abstract

Poly(glycerol sebacate) (PGS), a biodegradable elastomer, has been extensively explored in biomedical applications for its favorable mechanical properties and biocompatibility. Efforts have been made to fabricate multifunctional PGS copolymer in recent years, in particular PGS-*co*-PEG (poly(glycerol sebacate)-*co*-polyethylene glycol) polymers. However, rare research has been systematically conducted on the effect of reactant ratios on physicochemical properties and biocompatibility of PGS copolymer till now. In this study, a serial of PEGylated PGS (PEGS) with PEG content from 20% to 40% and carboxyl to hydroxyl from 0.67 to 2 were synthesized by thermal curing process. The effects of various PEGS on the mechanical strength and biological activity were further compared and optimized. The results showed that the PEGS elastomers around 20PEGS-1.0C/H and 40PEGS-1.5C/H exhibited the desirable hydrophilicity, degradation behaviors, mechanical properties and cell viability. Subsequently, the potential applications of the 20PEGS-1.0C/H and 40PEGS-1.5C/H in bone repair scaffold and vascular reconstruction were investigated and the results showed that 20PEGS-1.0C/H and 40PEGS-1.5C/H could significantly improve the mechanical strength for the calcium phosphate scaffolds and exhibited preferable molding capability for fabrication of the vascular substitute. These results confirmed that the optimized PEGS elastomers should be promising multifunctional substrates in biomedical applications.

## 1. Introduction

Synthetic biodegradable polymers, such as protein-like polypeptides [1,2], network polyesters [3,4] and hydrogels [5,6] have aroused great interest in various fields of biomedical engineering ranging from tissue regeneration to drug delivery platforms [7,8,9,10,11]. Among them, Poly(glycerol sebacate) (PGS), as a crosslinked biodegradable elastomer composed with two FDA-approved components, glycerol and sebacic acid, has been intensively leveraged in the tissue engineering due to its prominent biocompatibility and mechanical merits [12,13,14,15]. In recent decade, PGS-based blends have been further developed for tailored properties of PGS substrate and personalized tissue engineering applications via incorporating different functional agents or groups. For example, incorporation of conductive multiwall carbon nanotubes (MWSCNTs) into PGS rendered the increased matrix crosslinking and mechanical toughness and maintained its original flexibility [16,17]. Also, ultrathin two-dimensional (2D) nanosilicates were applied to improve mechanical strength and bioactivity of traditional PGS elastomers [18]. Besides, porous elastomeric grafts composed of PGS and PCL nanofibers have been investigated to promote the degradation behavior in arterial remodeling [19]. However, such PGS composites by a physical blend approach are prone to phase separation and thus triggering inhomogeneity and instability of the properties such as mechanical stiffness and bioactivity [20].

PGS-based copolymeric system was another strategy to improve the properties of PGS substrate. As previous reported, lactic acid was incorporated in to the PGS backbone for the purpose of increasing the molding capability and mechanical performance [21,22,23]. Meanwhile, glycolic acid was successfully segmented into PGS to accelerate the degradation rate [21,23]. Notably, these copolymeric elastomers usually exhibited mono functionality on tailoring degradation behavior or the mechanical properties of PGS. Polyethylene glycol (PEG), as an inert anionic and hydrophilic polymer is reckoned an ideal candidate for improving hydrophilicity and bringing about better cytocompatibility [24]. For instance, silk fibroin (SF) combined with the structural versatility of polyethylene-glycol-diacrylated (PEGDa) was produced for 3D-cell culture and showed potential as stem cell-carrier systems [25]. Recently, PEG as a hydrophilic segment that was widely utilized in amphiphilic and biodegradable block copolymers, have also been used for PGS modification [13,26]. PGS-*co*-PEG polymers could significantly tune the hydrophilicity of PGS while maintaining the controlled degradation behavior via altering the molar ratios of glycerol to PEG according to the previous research [27,28,29,30,31]. Such PEGS has been explored to coat onto the calcium phosphate cement (CPC) scaffold and the inspiring results manifested that PEGS coating could not only realize 5-fold mechanical enhancement, but also facilitate cell attachment and proliferation and further strongly promote the osteogenesis [32]. Despite successful synthesis and merits of PEGS in applications, work flow of PEGS has been refrained by multifactor including crosslinking time, PEG content and ratio of carboxyl to hydroxyl in reaction, etc. PEGS as a synthetic copolymer with excellent potential in biomedical applications and the optimization of its synthesis is of great importance. Unfortunately, to date, current researches seldom concentrated on the detailed work flow of PGS-related copolymers.

Herein, to optimize the properties of PEGS elastomer, a series of PEGS were synthesized with PEG content from 20% to 40% and 4 ratios of carboxyl to hydroxyl. And the effect of the dual variables of PEG addition and C/H ratio on the physicochemical and biological properties were assessed. The biocompatibility was evaluated through in vitro cell culture experiment. Finally, the optimized PEGS elastomers were explored for their latent utilization in vascular reconstruction and scaffolding modification applications.

## 2. Materials and Methods

### 2.1. Materials

The main reagents used for the polymerizations were purchased from Aldrich, Germany. These were sebacic acid (99.0% purity, Aladdin), glycerol (99.0% purity, Sigma-Aldrich), PEG (Aladdin 1000 g/mol). Ar (99.99% purity, Air Liquid) were used as received, ethanol (99.7% purity), dichloromethane (99.5% purity), chloroform-D (99.8% purity) and tris-(hydroxymethyl)-aminomethane solution (pH 7.4) (Tris–HCl, 99.0% purity) were all bought from Sinopharm Chemical Reagent Co., Ltd., (Shanghai, China). Dimethyl sulfoxide (DMSO) and 3-(4,5-dimethylthiazol-2-yl)-2,5-diphenyltetra-zolium bromide (MTT) were purchased from Sigma-Aldrich, (St. Louis, MO, USA). All other reagents used in cell culture were from Gibco (Grand Island, NY, USA).

### 2.2. Synthesis of PEGS Prepolymers and Elastomers

The synthesis of PEGS pre-polymers was performed via two-step polycondensation reaction between glycerol and sebacate. Two PEG content (20% and 40%) with four carboxyl/hydroxyl ratios (coded as C/H below), 2/3, 1/1, 3/2 and 2/1 respectively, were used to prepare PEGS elastomers of different cross-linking densities and compositions. The polymerization procedure for the synthesis of a certain elastomer with PEG content of 20% and C/H molar ratio of 1:1 was taken as an example for detailed description.

In the first step of the reaction, 0.02 mol PEG (Aladdin, MWs = 1000 g/mol) was dried to be molten and then mixed with 0.05 mol sebacic acid at 13 °C under the flow of Argon for 2 h before reacting under vacuum for another 24 h. In the second step, a specific amount of 0.08 mol glycerol and 0.09 mol sebacic acid was added into the flask with the flow of Argon until the mixture became clarified, then the reaction was further carried out at 130 °C and under vacuum for 48 h. The overall carboxyl to hydroxyl molar ratio was kept as 1:1. The obtained viscous mixture was dissolved in ethanol and purified by ultrapure water. The pre-polymer was obtained after dialyzing by 1000 D dialysis bag and freeze-dried in the lyophilizer. The cross-linked elastomers were produced in the desired shape by melting the prepolymer again at 130 °C, then casting it on a PTFE mold (10 mm × 5 mm × 1 mm), and placing it in a vacuum oven maintained at 150 °C for 36 h. The feed ratios of other PESG groups were listed in Table 1. All samples were coded as 20PEGS-0.67C/H, 20PEGS-1.0C/H, 20PEGS-1.5C/H, 20PEGS-2.0C/H, 40PEGS-0.67C/H, 40PEGS-1.0C/H, 40PEGS-1.5C/H and 40PEGS-2.0C/H respectively.

### 2.3. Characterization of the PEGS Prepolymer and Elastomer

#### 2.3.1. Gel Permeation Chromatograph (GPC) of Prepolymer PEGS

The molecular weights (MWs) and the molecular weights distributions (MWDs) of the PEGS prepolymers were characterized by gel permeation chromatography (GPC) using a Shimadzu Prominence HPLC instrument (Kyoto, Japan). The mobile phase was tetrahydrofuran (THF) and the samples was dissolved at 1 mg/mL in THF. The calibration curve was based on polyethylene glycol. For each sample, the following quantities were calculated from GPC: the number-average MWs, Mn, the polydispersity index (PDI = MWs/Mn, where MWs is the weight-average), and Mp is the MWs at the peak maximum.

#### 2.3.2. Fourier Transform Infrared (FT-IR) Spectra of Prepolymer PEGS

Fourier transform infrared (FT-IR) spectra (Nicolet 5700, Thermo) of 20PEGS and 40PEGS prepolymers were scanned in the range of 800–4000 cm^−1^ with a resolution of 4 cm^−1^. The spectra was obtained through touching the ATR objective on the samples and the data generated from the surface of the sample. The absorbance spectra was obtained with 16 scans per sample.

#### 2.3.3. Nuclear Magnetic Resonance (NMR) of Prepolymer PEGS

The components of 20PEGS and 40PEGS prepolymers were characterized by nuclear magnetic resonance (NMR) (Brukeravance II 600, Bruker Corporation, Zurich, Switzerland). The 20PEGS and 40PEGS prepolymers were firstly purified via precipitation in deionized water, and then were filtered and freeze-dried. The prepolymers were dissolved in deuterated chloroform (CDCl_3_) and the solution was subsequently transferred in NMR tubes. The resulting data were processed and analyzed using MestReNova NMR software.

### 2.4. Elastomer Characterization

#### 2.4.1. Hydrophilicity of PEGS Elastomers

The hydrophilic properties of PEGS with different proportions were characterized by contact angles of static water in air via Phoenix 300 contact angle measuring instrument. The cured PEGS elastomers were cut into square samples with a thickness of 0.5 mm and a side length of 10 mm. Droplets of ultrapure water with a volume of 4 μL were extruded onto the surface of the samples. The final contact angle was obtained by analyzing the tangent angle between the water droplets and the surface of the material. The images of the droplets on the samples were shot with a CCD camera, and the values of contact angle were assessed by image acquisition system.

#### 2.4.2. Gel Content of PEGS Elastomers

The crosslinking degree was manifested by the gel content. The characterization was carried out by analyzing the gel content of PEGS. *W_i_* g PEGS were immersed in THF. Three days later, the sample was taken out and dried to a constant weight. For each sample, five specimens were carried out and the results were averaged. After the THF was completely removed, the mass was weighed and recorded as *W_d_*. Formula (1) was used to calculate the sol content.

(1) $$\mathrm{Sol}\;\mathrm{Content}\;\left(\%\right)=\frac{W_{d}}{W_{i}}\times 100$$

#### 2.4.3. Mechanical Properties of PEGS Elastomers

The 20PEGS and 40PEGS prepolymers were melted and poured into the mold (10 mm × 5 mm × 1 mm), and the PEGS films were obtained by thermal curing. The obtained PEGS film was cut into a standard dumbbell sample (8 mm × 4 mm × 1 mm) with a standard cutter. The thickness of the sample was measured using a screw micrometer with an accuracy of 0.001 mm. After recording the size information of each sample, the upper and lower ends of the dumbbell sample were clamped with a universal testing machine (AG-2000A, Shimadzu, Kyoto, Japan), and the sample was stretched at a tensile speed of 50 mm/min. For each sample, five specimens were tested and repeated for three times and the results were averaged.

#### 2.4.4. In Vitro and In Vivo Degradation of PEGS Elastomers

The degradation performance was determined in accordance with the international standard (ISO 10993-14:2001, IDT). A Tris-HCl buffer solution with pH of 8.0 was prepared. The initial mass of PEGS sample was weighed as *W*_1_, and the prepared samples were immersed in Tris-HCl buffer according to the 1:10 ratio of sample mass (g) to solution volume (mL). The Tris-HCl solution for degradation should be changed once a day to simulate the circulation of body fluids. Samples at each time point were collected, vacuum dried and freeze-dried, and the mass was weighed as *W*_2_. For each sample, six specimens were tested, and the results were averaged. Formula (2) was used to calculate the residual mass of the material at each time point:

(2) $$\mathrm{Weight}\;\mathrm{Loss}\;\left(\%\right)\;=\frac{W_{1}-W_{2}}{W_{1}}\times 100$$

In order to further verify the degradation behavior of 20PEGS and 40PEGS in vivo, C57 mice were used as the model to carry out the subcutaneous implantation experiment on both sides of the back of mice under the condition of complying with the animal ethics and use guidelines set by the animal research ethics committee of East China university of Science and Technology. The eight-week-old male C57 mice were injected with 1% pentobarbital sodium (0.12 mL/g) in the abdominal cavity for anesthesia. After eliminating hair on back, the mouse was fixed on the ultra-clean bench with alcohol cotton disinfection. Surgical scissors were used to make about 6 mm incisions on both sides of the mouse back, and subcutaneous fascia was cut off at the same time. PEGS membrane sized 5 mm in diameter and 1 mm in thickness (weighed as *W*_3_) was implanted. When the predetermined time point was reached, a corresponding number of mice were injected and killed by excessive anesthesia. The sample with tissue was taken out, and the tissue was cut off, washed with ultra-pure water, and weighed *W*_4_ after freeze-drying. For each sample, six specimens were tested and the results were averaged. Finally, the in vivo degradation rate of PEGS was obtained by Formula (3).

(3) $$\mathrm{Weight}\;\mathrm{Loss}\;\left(\%\right)\;=\frac{W_{3}-W_{4}}{W_{3}}\times 100$$

### 2.5. In Vitro Cell Culture Experiments

#### Cell Viability of PEGS Elastomers

The Mesenchymal stem cells (MSCs) isolated from rat bone marrow, were cultivated in α-MEM medium supplemented with 10% fetal bovine serum (FBS) (HyCloneTM) at 37 °C in a humidified atmosphere containing 5% CO_2_. The medium was refreshed every other day. The HUVECs were placed in a 25 cm^2^ tissue culture polystyrene flask and incubated at 37 °C in a humidified atmosphere containing 5% CO_2_. The culture medium was also refreshed every other day. Before seeding, PEGS elastomer membrane was sterilized and then put in a 48-well tissue culture plate. For all experiments, cells between the 2nd and 4th generation were used and seeded on materials surface with a density of 5000 cells per well, and cultured for 1, 3 and 7 days in growth medium. Cell viability was evaluated by MTT assay [33]. At predetermined culture time, each well was treated with MTT solution and incubated at 37 °C for 4 h, followed by DMSO dissolving. Tissue culture plastic (TCP) served as positive control.

### 2.6. Optimal PEGS Elastomers in Biomedical Application

#### 2.6.1. Fabrication of PEGS/Calcium Phosphate Scaffold

Calcium phosphate (CaP) scaffolds were prepared with equimolar tetracalcium phosphate (TTCP, Ca_4_(PO_4_)_2_O) and dicalcium phosphate anhydrous (DCPA, CaHPO_4_), in which NaCl granules with 300–500-μm size were incorporated and mixed with 100 μL saturated NaCl solution in H_2_O while stirring to form the cement slurry. The slurry was then cast into the mold under a pressure of 2 MPa for 1 min to prepare cylinder samples (Ø10 × 5mm). After 3 days of curing in the condition of 100% air humidity and 37 °C, the samples were soaked in purified water for 2 days until the completely dissolution of NaCl granules and then dried at 100 °C for 12 h to obtain the porous CaP scaffolds. For PEGS, 20PEGS-1.0C/H and 40PEGS-1.5C/H prepolymers were dissolved in ethanol at a mass/volume ratio of 0.4 g/mL with the use of ultra-sonotor to form a homogeneous solution. These solutions were dropped and infiltrated into CaP scaffolds, in batches, at 10 μL/cm^2^ using a pipettor, with time interval between batches for complete penetration and uniform distribution. And then, the PEGS/phosphate scaffolds were treated at 150 °C for 36 h in the vacuum oven for further curing.

#### 2.6.2. Mechanical Properties of PEGS/Calcium Phosphate Scaffold

Mechanical strength of PEGS/CaP hybrid scaffold was measured using a universal testing machine (AG-2000A, Shimadzu, Kyoto, Japan) with steel discoid indenters. Compressive strength could be defined as ultimate compressive stress when the specimen fracture. For each group, five specimens were tested, and the results were averaged.

#### 2.6.3. Artificial Vascular Reconstruction

A vascular mold was customized with the size close to the natural artery vessel. The 20PEGS-1.0C/H and 40PEGS-1.5C/H prepolymers were then melted and poured into the mold. Together with the mold, the prepolymers were transferred into a vacuum drying oven and cured at 150 °C for 36 h.

### 2.7. Statistical Analysis

All data were expressed with mean standard deviation (SD) and analyzed using one-way ANOVA followed by Tukey’s test. A statistical significance was accepted at * *p* < 0.05, ** *p* < 0.01, and *** *p* < 0.001.

## 3. Results and Discussion

### 3.1. Synthesis and Characterization of PEGS Prepolymers

**Figure 1:**
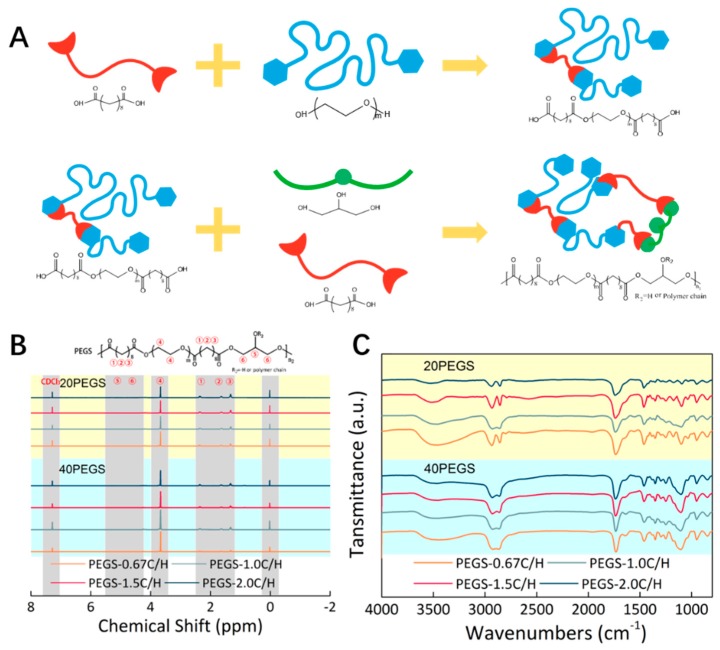
Synthetic process and characterization of PEGS prepolymer. (**A**) Prepolymer PEGS was synthesized by specific molar ratio via two-step polycondensation. (**B**) ^1^H-NMR spectra of prepolymer PEGS. (**C**) FT-IR spectra of prepolymer PEGS.

Branched PEGS20 and PEGS40 were synthesized via two-step polycondensation with controlled feeding as shown in Figure 1A, sebacic acid and PEG were copolymerized in the first step followed by the copolymerization with glycerol and rest sebacic acid in the second step. Through altering PEG content and ratio of carboxyl to hydroxyl, a series of PEGS polymers were designed and synthesized.

The successful synthesis and molecular structure of the PEGS derivatives were directly characterized by FTIR and ^1^H–NMR spectroscopy. As shown in Figure 1B, the observed peaks near 1150 cm^−1^ were corresponding to the ether bond denoting PEG was segmented successfully into the PEGS backbone. Typical broad peaks around 3500 cm^−1^ resulting from the stretching vibration of free hydroxyl groups were presented among all the 20PEGS and 40PEGS groups. The reduction in the hydroxyl at 3500 cm^−1^ occurred with the increase of PEG segment. Additionally, more introduction of PEG segments for caused an increase in the ratio of methylene peak to hydroxyl peak from 5.92 of 20PEGS group to 7.33 of 40PEGS group. To be noted, slight shift of the characteristic absorption peak of hydroxyl in the direction of lower wavenumbers was observed between 20PEGS groups and 40PEGS groups which could be explained by the hydrogen bond bilaterally. On one hand, the increased PEG content enhanced the hydrogen bond effect, on the other hand, the lower C/H ratio led to lifted amount of hydroxyl groups in the backbone of PEGS. The peaks near 1750 cm^−1^ derived from the stretching vibration of carbonyl groups implied the esterification reaction between sebacic acid, glycerol and PEG, which was aligned with the previous report [12].

Detailed composition of 20PEGS and 40PEGS prepolymer was further confirmed through ^1^H–NMR spectroscopy. As is shown in Figure 1C, three proton peaks were observed at δ 1.30, 1.62 and 2.34 ppm corelated to the sebacoyl moiety in the PEGS backbone, and the peaks at δ 3.56–4.41 ppm and 4.90–5.30 ppm corelated to the glycerol moiety in the PEGS chain as previous studies [12]. The incorporation of PEG segment was affirmed according to the presence of methylene peak emerging in the spectra of both 20PEGS and 40PEGS at 3.77 ppm [13]. The quantitative analysis of the actual PEG content and proportion of carboxyl and hydroxyl was based on the calculation of NMR data and listed in Table 2 along with the theoretical estimation. The actual molar composition was proved in good agreement with the theoretical estimation in the groups with ratio of carboxyl to hydroxyl fixed at 1.0 and 1.5. However, deviations from the theoretical values happened in the 20PEGS-0.67CH and 40PEGS-0.67CH group whose actual C/H was nearly 1.0. This could be possibly ascribed to the equimolar reaction mechanism in the condensation polymerization, which meant the excessive glycerol might not undergo further reaction and resided in the form of monomer rather than incorporating into the polymeric chain, thus increasing the actual ratio of carboxyl to hydroxyl and PEG content [29]. Despite equimolar reaction mechanism, it’s worth noting that according to previous research, the excessive carboxyl tended to be completely reacted as it could promote the crosslinking of pre-PEGS [13].

Next, we detected the molecular weights of various PEGS prepolymers by GPC; the results are listed in Table 3. The results exhibited relatively low molecular weight from 3213 Da to 11,516 Da of both 20PEGS and 40PEGS prepolymers, indicating low viscosity and favorable liquidity of PGES solution in solvent (for further modification in other application). In comparison, the increase of PEG content reduced the molecular weight. This phenomenon could be ascribed to the declined proportion of glycerol and sebacic acid monomer in the whole reaction. Moreover, PEG as relatively more structured segment in the PEGS polymer chain decreased the crystallinity, implying the lower molecular weight of 40PEGS groups than that of 20PEGS groups. According to the feed ratio, doubled PEG incorporation might halve the feeding of glycerol and sebacic acid monomer leading to a lower MWs [13]. Furthermore, in both 20PEGS and 40PEGS, the molecular weight underwent the same trend of rise and fall along with the incremental C/H ratio because of the facet of stoichiometric. For PDI, appropriate increase of C/H ratio may decrease the PDI. More importantly, the mechanism of PEGS reaction largely resembled 3-2 polycondensation of PGS where PDI was susceptible to the branched chain [29]. The PEG segment introduced into the main chain indirectly lowered the side chain proportion and reduced the branching index thus lowered the overall PDI and consequently led to more harsh reaction condition for the next crosslinking.

### 3.2. Gel Fraction of PEGS Elastomers

Generally, conventional PGS was crosslinked at around 120 to 130 °C [12,32], however PEG segment indirectly reduced content of hydroxyl in the PEGS backbone and hereby raised reaction barriers [29,34]. Meanwhile, although prolonging reaction time favored for further crosslinking, the polycondensation kinetics implied less assistance to the reaction process, even might impair the stability of the PEGS. Hence, raising the temperature was of highest priority and three temperature was investigated for the crosslinking behavior with the reaction time set as 12 h and 36 h.

**Figure 2:**
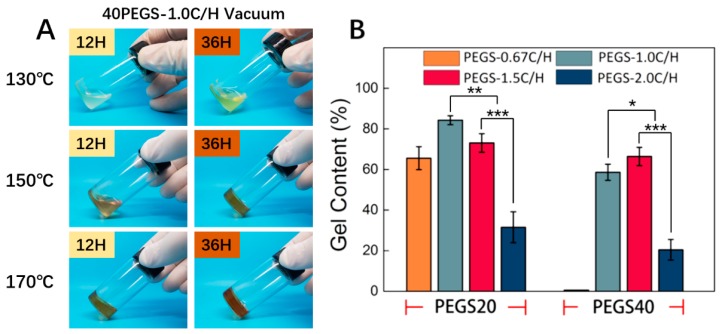
Curing process and crosslinking density of PEGS. (**A**) Prepolymer PEGS was cured at 130, 150 and 170 °C for 12 h and 36 h. (**B**) Gel content of PEGS elastomers cured at 150 °C. Asterisks indicate significant differences, * *p* < 0.05, ** *p* < 0.01, *** *p* < 0.005.

During the curing course at high temperature, the prepolymer PEGS went through a sol-gel transition along with the change of color. In consideration of the residual oxygen, PEG incorporated into the backbone of PGS was vulnerable to oxidization under high temperature and simultaneously presented yellow appearance. As the reaction progress, the white transparent PEGS prepolymer gradually turned into semifluid state with yellowing color and ultimately cured with color deepened. As shown in Figure 2A, 40PEGS-1.0C/H as example, when the temperature was set at 130 °C, the viscosity of the PEGS increased but the PEGS failed to crosslink. With the temperature raised to 170 °C, the PEGS partially crosslinked at early 12 h and the color was continuously deepened at 36 h suggesting the severe oxidization of PEG. The byproduct of oxidized PEG would affect the cell mortality [35]. Under 150 °C, the PEGS remained viscous status at 12 h and crosslinked at 36 h with the color acceptable. To strike the balance between the crosslinking time and oxidization, 150 °C was finally chosen as the crosslinking temperature in the following section.

Then gel content of PEGS after curing was analyzed at 150 °C to determine the crosslinking degree of PEGS. It is well acknowledged that chemical composition was one of the most important factors deciding the crosslinking behavior. Figure 2B shows the gel content of PEGS with different proportions after thermal cross-linking. Among all the PEGS groups, the result of gel content displayed similar correlation with C/H ratio between 20PEGS and 40PEGS groups, except for the 40PEGS-0.67C/H group due to its non-crosslinking consequence under the set reaction condition. The gel content in all 20PEGS and three of 40PEGS cases widely ranged between 20% and 83% and PEG content displayed subtle influence on the result. There were two kinds of hydroxyl in the glycerol reacting during the esterification, nevertheless, primary hydroxyl accounted for the chain growth, only para ones played the key role in the sequential crosslinking. Notably, during the crosslinking, the para hydroxyl was severely affected by the steric hinderance and embedded effect of polymeric chain which hampered the crosslinking rate. The higher C/H ratio provided more carboxyl to attack the para hydroxyl after chain growth thus directly promoting the crosslinking procedure. Besides, for the 1.5C/H group, the excessive carboxyl group resulted in partial cross-linking of PEGS prepolymer during the synthesis process, which was also indicated by the increased PDI. For the 40PEGS groups, the gel content showed adverse effect when considering C/H ratio. Despite the slight deviation from the stoichiometry, excessive carboxyl in 40PEGS-1.5C/H could meet the demanding requirement for the tougher crosslinking when the PEG content increased. What’s more, doubled content of PEG introduction lowered the relative amount of hydroxyl in the polymer backbone, therefore higher concentration of carboxyl or temperature was needed. For the 20PEGS groups, since the stoichiometric ratio of 1.0C/H group was 1, the reaction endured higher degree of esterification and eventually yielded the prepolymer with greater MWs. The less gel content was observed for the 20PESG-0.67C/H and 2.0C/H groups whose C/H ratio was away from stoichiometric and MWs were relatively low, it therefore had the lower cross-linking density. The lowest gel content in PEGS-2.0C/H was attributed to the least esterification caused by the excessive amount of unreacted sebacic acid monomer. Since the unsatisfactory crosslinking density may inevitably hamper the mechanical properties and uncontrollable degradation, the 20PEGS-2.0C/H and 40PEGS-2.0C/H will be excluded in the following study.

### 3.3. Mechanical Properties of PEGS Elastomers

**Figure 3:**
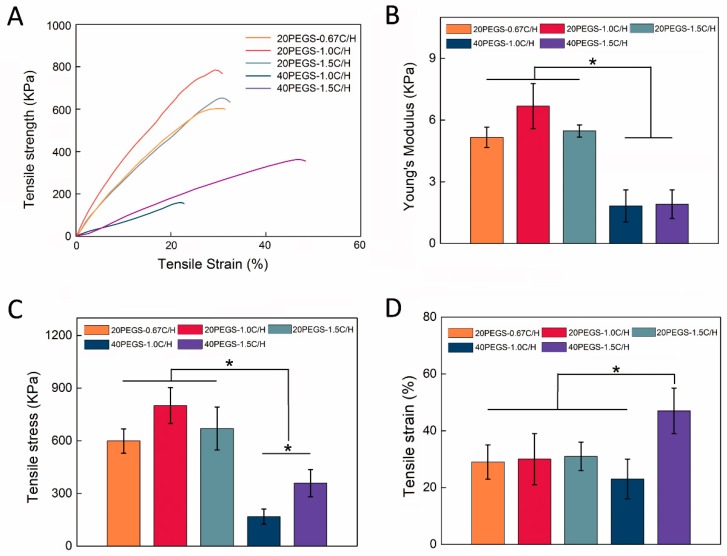
Mechanical properties of PEGS. (**A**) Tensile stress-strain curves of PEGS elastomers. (**B**) Young’s modulus, (**C**) Tensile stress and (**D**) Tensile strain of PEGS elastomers. Asterisks indicate significant differences, * *p* < 0.05.

As earlier studies reported, tensile strength of PGS and its derivatives have exhibited that the material presented nonlinear stress–strain behavior, which is typical for soft elastomeric materials [36]. In previous research, the addition of PEG into the backbone of PGS was found to greatly enhance the elastomeric properties, which revealed that PEGS could be subjected to extreme deformation without fraction. To better elucidate the effect of diverse reactant condition on the mechanical properties of PEGylated polymers including Young’s modulus (YM), ultimate stress and ultimate elongation, five PEGS groups, which were able to be crosslinked to form tough elastomer, tensile tests were carried out on the prepared samples. As expected, all PEGS samples showed a decreased tensile strength but significantly higher tensile strain than conventional PGS elastomer as previous reported [13]. As shown in Figure 3A, the mechanical strength of 40PEGS group was obviously lower than that of 20PEGS, mainly because more introduction of PEG flexible chain segment reduced the percentage of rigid chain segments in the PEGS, and at the same time reduced the crosslinking density [37]. Nevertheless, 40PEGS tended to increase on elongation ascribed to the PEG flexible chain segments.

The specific data for the mechanical properties of PEGS were shown in Table 4. For the 20PEGS group, as shown in Figure 3A, the ultimate tensile strength (UTS) ranked as 20PEGS-1.0C/H>20PEGS-1.5C/H>20PEGS-0.67C/H, ranging from 599 to 801 KPa as shown in Figure 3C, while YM ranged from 516 to 668 KPa as shown in Figure 3B, which could be jointly determined by the crosslinking degree and molecular weight to some extent. When the MWs went up, the PEGS chain was prone to be more entangled bringing about stronger resistance to the chain slip caused by external stress on the molecular chain. Consequently, 20PEGS-1.0C/H and 20PEGS-1.5C/H with higher MWs exhibited more favorable mechanical strength than 20PEGS-0.67C/H. As shown in Figure 3D, in terms of elongation at break, there was no significant difference among the three proportions in the 20PEGS group, indicating that the change of the C/H ratio affected less on this aspect. In the 40PEGS group, UTS and YM showed a sharp decrease compared to the 20PEGS group, with the UTS ranging from 168 to 359 KPa and YM ranging from 181 to 193 KPa, indicating that PEG content plays a leading role in mechanical performance. It is worth noting that the elongation at break of 40PEGS-1.5C/H almost doubled that of 20PEGS, but the elongation at break of 40PEGS-1.0C/H was not enhanced so. Since the 40PEGS-1.5C/H obtained increased crosslinking density via the excessive sebacic acid, the relative MWs of the chain segment between the crosslinking nodes was increased along with the chain getting more entangled, eventually, the elongation at break presented doubled performance.

### 3.4. Hydrophilicity and Degradation of PEGS Elastomers

**Figure 4:**
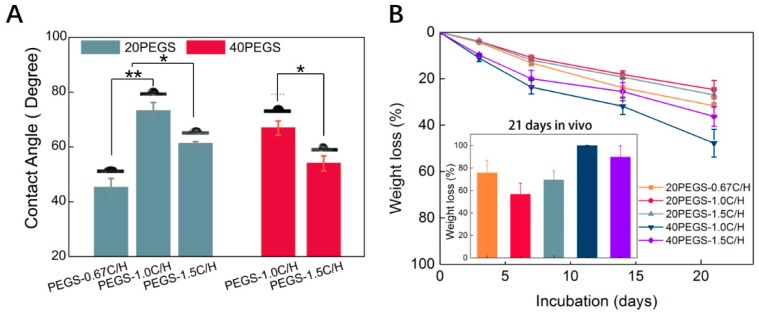
Hydrophilicity and degradation behavior of PEGS elastomers. (**A**) Contact angle of PEGS elastomers. (**B**) In vitro and in vivo degradation of PEGS elastomers. Asterisks indicate significant differences, * *p* < 0.05, ** *p* < 0.01.

As is one of the key factors for degradation and biocompatibility, knowledge of the hydrophilicity of PEGS elastomers is of necessity for biomedical applications. The hydrophilicity of the elastomer could be improved via the covalent incorporation of PEG and tuned through adjustment of PEG content and C/H ratio during synthesis. As shown in Figure 4A, with the increase of PEG content, the contact angle reduced from 75.5° ± 2.5° of 20PEGS-1.0C/H to 68.4° ± 2.3° of 40PEGS-1.0C/H. When the C/H ratio went up to 1.5, the variation of contact angle was in line with 1.0C/H groups. Since the contact angle is closely correlated to the MWs and crosslinking density, it’s worth noting that compared to the hydrophilicity lifted by PEG, the C/H ratio had a greater impact on the regulation of hydrophilicity. In 20PEGS group, the 20PEGS-0.67C/H showed the smallest contact angle of 48.4° ± 2.6°, followed by 20PEGS-1.5C/H group of 61.3° ± 0.6° while the largest is the 20PEGS-1.0C/H and the 40PEGS group shared the same tendency.

Controlled degradation is a significant factor for bio-elastomers, greatly determining their biomedical application in tissue engineering. Most bio-elastomers faced the mismatching of degradation rate to the tissue formation and were limited in tissue regeneration [38,39,40]. Generally, the degradation is determined by many factors, including molecular weight, crosslinking degree and hydrophilicity. Therefore, PEGS prepared by different proportions also exhibited different degradation performance. Based on degradation mechanism of ester bond hydrolysis for PEGS, Figure 4B showed the in vitro and in vivo degradation of 20PEGS and 40PEGS within 21 days. PEG, as hydrophilic segment, was introduced to promote the hydrophilicity therefore 40PEGS group showed faster degradation rate than 20PEGS group both in vitro and in vivo [13]. In the 20PEGS group, determined by molecular weight and crosslinking degree, the degradation rate was ranked as 20PEGS-0.67C/H>20PEGS-1.5C/H>20PEGS-1.0C/H. Despite the similar molecular weights of 20PEGS-1.0C/H and 20PEGS-1.5C/H, the degradation of 20PEGS-1.0C/H was a bit slower due to the higher crosslinking degree. The overall degradation rate of 20PEGS was in the same trend with the crosslinking degree and hydrophilicity. For the 40PEGS group, the molecular weight of 40PEGS-1.0C/H was much lower than that of 40 PEGS-1.5C/H and the gel content was relatively lower. Therefore, 40 PEGS-1.0C/H showed a faster degradation rate. The degradation performance of biomaterials is an important index of tissue regeneration based on scaffold substrate, and the appropriate degradation rate should match the growth rate of new tissues, so too fast or too slow degradation rate cannot meet the actual clinical needs. As shown in the results of subcutaneous administration, 40PEGS groups tended to degrade totally within 21 days in the body, which might lead to the loss of mechanical support and thereby cause secondary defect of implantation. From the point of stability during the repairing period, 20PEGS with relatively slow degradation performance revealed more advantages than 40PEGS.

### 3.5. In Vitro Cell Culture Evaluation

**Figure 5:**
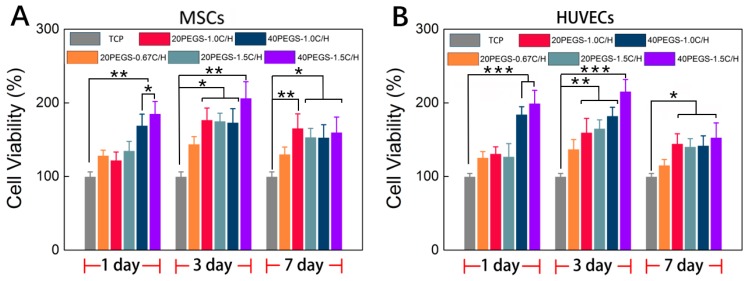
Biocompatibility of PEGS elastomers. (**A**) MSCs cell viability on PEGS elastomers. (**B**) HUVECs cell viability on PEGS elastomers, Asterisks indicate significant differences, * *p* < 0.05, ** *p* < 0.01, *** *p* < 0.005. vs. the corresponding tissue culture plate (TCP) group).

As one of the most paramount characteristics of bio-elastomers, biocompatibility determines the performance of host response when the materials was brought into contact with the surrounding tissue. According to the previous research [37,41], PEG incorporation could enhance the biocompatibility due to the increased hydrophilicity. As shown in Figure 5A, MSCs was seeded onto the PEGS membrane with different PEG content and C/H ratio, and the cell attachment and proliferation performance of MSCs were evaluated through MTT assay. The initial cell responses were well acknowledged to influence the ultimate biofunction. Through the quantitative cell viability evaluation, for the initial 24 h, all the samples were indicated nontoxicity in which 40PEGS groups possessed higher cell viability compared to 20PEGS groups indicating better cell attachment in the early stage. In the consistence with the results of previous research, the copolymerization of PEG dominantly raised the cell affinity via the improvement of the surface wettability which accounted for the better cell adhesion on 40PEGS group than 20PEGS group. Besides, the cell viability was also trimmed by the C/H ratio for 40PEGS-1.0C/H and 40PEGS-1.5C/H. Higher C/H ratio rendered improved hydrophilicity leading to better cell adhesion thus promoting the cell viability in the 40PEGS group while no significant difference was observed among the 20PEGS group. The proliferation of MSCs was further investigated for a longer period of 3 and 7 days. As is shown in Figure 5A, the cell viability of all the samples reached the maximum after 3-day incubation and underwent a minor fall after rise at day 7, indicating that hardly toxicity emerged in the late stage. The slight decline in proliferation happened at day 7 was probably ascribed to the acidified surrounding caused by degradation. Commonly, the synthetic polyester degradation started at the break of ester linkage. During this process, the degradation products gradually acidized the medium and further accelerated the degradation behavior after 7 days which induced suppressed proliferation and slight decline. But in vivo, by the uninterrupted fleeting metabolism, the acidized medium was neutralized, and negative effect was alleviated thus generating little impact on the cell fate in proliferation.

To further investigate the potential application of PEGS in vascular repair, HUVECs was also seeded onto the PEGS membrane, and the attachment and proliferation were evaluated via MTT assay as shown in Figure 5B. The cell viability manifested the same tendency of MSCs MTT result suggesting that PEGS elastomer could support HUVECs adhesion and proliferation, thus extending the application in vascular repair.

### 3.6. PGES Biomedical Application

As one of the most potential applications of bio-elastomers, PEGS is good candidate in diverse biomedical application such as reinforcement phase for bone repair scaffolds and artificial vascular construction [13,32]. According to the previous study, PEGS could effectively improve the hydrophilicity and cell response of calcium phosphate scaffold as well as the mechanical properties [32]. Besides, PGS has been made into blood vessels, which were co-cultured with smooth muscle cells in a pulsed flow cell reactor, and the elastin content was about 20% of the natural blood vessels [1]. From the above, given the overall properties of PEGS with gradient PEG and C/H ratio, 20PEGS-1.0C/H and 40PEGS-1.5C/H exhibited optimal physiochemical properties and biocompatibility therefore being selected to carry out the following experiments.

**Figure 6:**
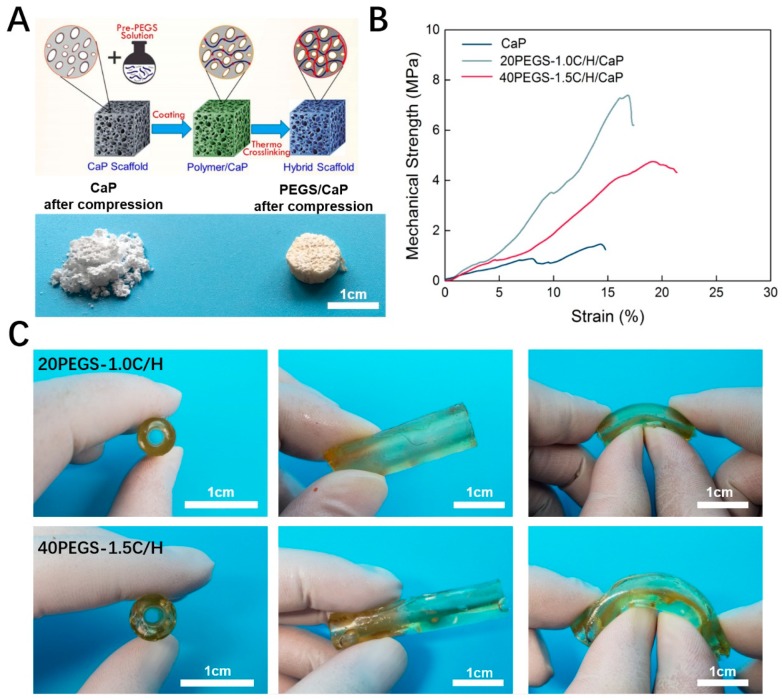
Biomedical application of PEGS. (**A**) PEGS/CaP composite scaffold with enhanced compression resistance (CaP and 20PEGS-1.0C/H/CaP for illustration). Scale bar: 1 cm (**B**) Compressive stress-strain curve of PEGS/CaP hybrid scaffolds. (**C**) Morphology of 20PEGS-1.0C/H and 40PEGS-1.5C/H tubes for potential vessel construction. Scale bar: 1 cm.

Recent years have witnessed a great deal of bone defects occurrence due to accidents, diseases, natural disasters and other incidents, therefore bringing about a great amount of bone defects patients. Calcium phosphate substrates such as hydroxyapatite (HAP) and β-tricalcium phosphate (β-TCP) have been widely used as scaffolds for bone tissue repair due to its good biocompatibility, biodegradability, osteoconductivity, little toxic side effects and other characteristics [42,43]. Previously, our group has successfully fabricated CaP-based bioceramics (mesoporous bioglass (MBG) and β-TCP) for effective enhancement of the toughness and the prepared MBG-modified β-TCP scaffold showed favorable biocompatibility and osteoconductivity [44,45,46]. However, the porous calcium phosphate scaffold prepared still exhibited undesirable mechanical properties, especially with high porosity, which greatly limited its clinical application [44]. Figure 6A showed the sponge template scheme of PEGS/phosphate composite scaffold fabrication, and the contrast before and after compression of CaP and PEGS/CaP scaffold. In this experiment, 20PEGS-1.0C/H and 40PEGS-1.5C/H were utilized to endow better mechanical performance for CaP scaffolds. It’s notable that through thermal curing, the PEGS-/CaP scaffolds displayed yellowish appearance and showed preferable resistance to compression compared to the traditional one which was crushed. Both PEGS/CaP hybrid scaffolds exhibited enhanced toughness whose maximum ultimate compression strength (UCS) reaching 7.6 ± 0.2 MPa for 20PEGS-1.0C/H/CaP group and 4.9 ± 0.3 MPa for 40PEGS-1.0C/H/CaP group, about 5 and 3 times that of pure CaP scaffold (1.5 ± 0.2 MPa) respectively as shown in Table 5. These results suggested that the PEGS coating would significantly improve the mechanical performance of CaP scaffold.

When comes to the vascular reconstruction, complicated shape of natural blood vessels has put forward high demand for the construction of substitute elastomer. In this study, by virtual of favorable fluidity of melt PEGS prepolymer, more diverse and convenient shaping was rendered which could be harnessed to reach the order for complex vascular reconstruction. As is shown in Figure 6C, the resultant tube showed homogeneous wall thickness (2 mm in inner diameter, 2 mm thick) and preferable tenacity proved both 20PEGS-1.0C/H and 40PEGS-1.5C/H to be desirable artificial vessel substitute for potential vessel construction.

## 4. Conclusions

In this study, prepolymer PEGS with different PEG content and C/H ratio were successfully synthesized and were further crosslinked by conventional thermal curing at 150 °C. The PEGS elastomers with different feeding ratios were obtained and optimized according to their physiochemical properties and biocompatibility. Optimal efficiency was observed around 20PEGS-1.0C/H and 40PEGS-1.5C/H, and showed complementary hydrophilicity, degradation behaviors, mechanical properties and cell viability. In detail, the 20PEGS-1.0C/H exhibited better mechanical strength as large as 801 ± 103 KPa in UTS and 668 ± 111 KPa in a Young’s modulus in sacrifice of a little hydrophilicity and degradation rate. On the contrary, 40PEGS-1.5C/H showed enhanced hydrophilicity with contact angle of 56.2° ± 2.1° and accelerated degradation rate with 90% weight loss within 21 days at the cost of mechanical strength. The optimized PEGS were further explored for the application in bone repair scaffold and vascular fabrication. Enhanced mechanical strength of CaP scaffold and favorable molding capability of artificial vessel provided strong evidence that the optimized PEGS elastomers should be promising candidates for various biomedical application.

## Figures and Tables

**Table 1:** Feed ratio of the preparation of prepolymer PEGS.

Pre-Polymer	Sample Code	Carboxyl/Hydroxyl	Feed
**20PEGS**	20PEGS-0.67C/H	2/3	Glycerol 0.08 mol, PEG 0.02 mol, sebacic acid 0.093 mol
	20PEGS-1.0C/H	1/1	Glycerol 0.08 mol, PEG 0.02 mol, sebacic acid 0.14 mol
	20PEGS-1.5C/H	3/2	Glycerol 0.08 mol, PEG 0.02 mol, sebacic acid 0.21 mol
	20PEGS-2.0C/H	2/1	Glycerol 0.08 mol, PEG 0.02 mol, sebacic acid 0.28 mol
**40PEGS**	40PEGS-0.67C/H	2/3	Glycerol 0.03 mol, PEG 0.02 mol, sebacic acid0.045 mol
	40PEGS-1.0C/H	1/1	Glycerol0.03 mol, PEG 0.02 mol, sebacic acid 0.065 mol
	40PEGS-1.5C/H	3/2	Glycerol 0.03 mol, PEG 0.02 mol, sebacic acid 0.0975 mol
	40PEGS-2.0C/H	2/1	Glycerol 0.03 mol, PEG 0.02 mol, sebacic acid 0.13 mol

**Table 2:** Theoretical and actual ratio of prepolymer PEGS.

Sample	PEG Content %		Ratio (COOH/OH)	
	By ^1^H–NMR	Theoretical	By ^1^H–NMR	Theoretical
20PEGS-0.67C/H	20.89	20	1.97/2.00	2.0/3.0
20PEGS-1.0C/H	17.40	20	0.97/1.00	1.0/1.0
20PEGS-1.5C/H	19.08	20	3.00/2.03	3.0/2.0
20PEGS-2.0C/H	15.47	20	2.00/1.21	2.0/1.0
40PEGS-0.67C/H	48.56	40	1.92/2.00	2.0/3.0
40PEGS-1.0C/H	39.08	40	0.91/1.00	1.0/1.0
40PEGS-1.5C/H	39.93	40	3.00/1.82	3.0/2.0
40PEGS-2.0C/H	37.38	40	2.00/1.01	2.0/1.0

**Table 3:** GPC result of prepolymer PEGS.

Distribution Name		Mn (Daltons)	MWs (Daltons)	MP (Daltons)	PDI
20PEGS	PEGS-0.67C/H	4355	6966	6124	1.599
	PEGS-1.0C/H	5771	10,467	6577	1.814
	PEGS-1.5C/H	6203	11,516	6761	1.856
	PEGS-2.0C/H	4132	5548	2651	1.342
40PEGS	PEGS-0.67C/H	3264	4165	4931	1.276
	PEGS-1.0C/H	4620	6934	5099	1.501
	PEGS-1.5C/H	5575	9433	6075	1.692
	PEGS-2.0C/H	3068	3213	3677	1.047

**Table 4:** Mechanical properties of the PEGS elastomers.

Sample Code	Tensile Stress (KPa)	Tensile Strain (%)	YM(KPa)
20PEGS-0.67C/H	599 ± 69	29 ± 6	516 ± 53
20PEGS-1.0C/H	801 ± 103	30 ± 9	668 ± 111
20PEGS-1.5C/H	670 ± 122	31 ± 5	547 ± 37
40PEGS-1.0C/H	168 ± 43	23 ± 7	183 ± 78
40PEGS-1.5C/H	359 ± 77	47 ± 8	191 ± 79

**Table 5:** Mechanical properties of the PEGS/CaP scaffolds.

Sample Code	Mechanical Strength (MPa)	Strain (%)	YM(MPa)
CaP	1.5 ± 0.2	14.8 ± 0.7	3.2 ± 0.9
20PEGS-1.0C/H/CaP	7.6 ± 0.2	16.5 ± 0.9	14.7 ± 1.1
40PEGS-1.5C/H/CaP	4.9 ± 0.3	21.2 ± 0.5	7.4 ± 0.7
